# Edge-Optimized Semi-Supervised Deep Learning for Power Line Component Inspection †

**DOI:** 10.3390/s26133969

**Published:** 2026-06-23

**Authors:** Nico Surantha, Hanfei Zhang, Daiki Watanabe

**Affiliations:** Department of Electrical, Electronics, and Communication Engineering, Faculty of Science and Engineering, Tokyo City University, Tokyo 158-8557, Japan

**Keywords:** edge-AI, YOLO, Jetson Orin Nano, Raspberry Pi 5, semi-supervised learning

## Abstract

Power line component inspection is essential for maintaining the reliability of the electrical power infrastructure. Recently, some researchers have studied automatic power line inspection using drones and deep learning. However, fully supervised deep learning approaches require large amounts of labeled data that are difficult and expensive to obtain in real-world environments. To address these challenges, this paper proposes an edge-optimized semi-supervised deep learning framework for power line component inspection. The proposed approach combines a semi-supervised learning (SSL) strategy to leverage both limited labeled images and abundant unlabeled field data with hardware–software (HW-SW) co-optimization techniques for efficient deployment on resource-constrained edge devices. In the learning stage, the framework improves detection performance by leveraging unlabeled inspection data via pseudo-labeling and confidence-based sample selection, thereby reducing annotation effort while maintaining robust recognition performance. In the deployment stage, the quantization technique was applied to enable real-time operation on embedded platforms with limited computational resources and power budgets. In this paper, an improved version of the edge-AI deployment score, the generalized edge-AI deployment score (GEADS), is proposed. In SSL evaluation, debiased semi-supervised learning (DeSSL) achieves a higher observed mAP@0.5 and F1-score than the standard SSL method in the single-run simulations using dataset 1 and dataset 2. In hardware evaluation, the YOLOv7-Tiny (INT8) configuration implemented on a Raspberry Pi 5 achieves the highest GEADS of 0.657, confirming it offers the most balanced performance among the required parameters. From the simulation, it is also confirmed that the proposed GEADS provides a more interpretable and statistically stable metric than the existing metric to evaluate the edge deployment.

## 1. Introduction

Ensuring the safety and reliability of the inspection process has become increasingly important as modernization and the swift expansion of worldwide power line networks have accelerated. Power line components are prone to damage from natural disasters such as fires, leaks, and lightning strikes. This damage can lead to problems such as overheating and arching [1]. Although traditional manual inspections can identify potential faults, they are labor-intensive, expensive, and prone to unpredictability due to operator expertise and environmental circumstances. Deep-learning-based automated inspection systems offer a promising approach for power line equipment surveillance due to their high detection accuracy, safety, and lower labor intensity [2].

To create a more effective, less risky solution for power line inspection, researchers recently began investigating automated inspection assisted by a drone [3]. The researchers have found it difficult to develop more reliable technology for this purpose. A drone system equipped with cameras and sensors can monitor power line equipment. However, it requires a portable computing system with artificial intelligence (AI) capabilities for automatic inspection. An edge-computing device is a portable computing system that incorporates microprocessors, memory, and a hardware accelerator. Edge-computing platforms, including Raspberry Pi [4], Google Coral [5], Jetson Nano [6], and FPGA-based systems like Kria KV-260 [7] are frequently utilized for AI processing on drones. One of the challenges in this research is to develop a solution that can achieve a balanced performance between accuracy, inference latency, resource utilization, and power consumption. Among deep learning methods, the You Only Look Once (YOLO) family stands out for its excellent trade-off between inference speed and detection precision [8], making it a top choice for real-time detection tasks. Our previous research proposed YOLOv3 in Raspberry Pi 4B [4]. Our previous results show that YOLOv3 can achieve acceptable detection performance but not real-time performance.

Another problem in developing drone-based power line inspection is the availability of sufficiently large, diverse, and well-annotated relevant datasets captured from real operating environments. STN PLAD explicitly notes that public data related to power line assets is rarely available [9]. Some of them do not cover enough power line components, while others are private due to the agreement with the institution or government agencies that funded the research, while InsPLAD similarly states that automatic power line visual inspection remains an open problem because of the lack of public real-world datasets covering both components and their defects. Even InsPLAD, although larger, contains 10,607 UAV images with 17 asset types and several defect types, which highlights how specialized and costly such data collection is [10]. In addition, InsPLAD emphasizes that real inspection images are affected by uncontrolled conditions such as multi-scale objects, cluttered backgrounds, viewpoint variation, occlusion, and lighting changes, all of which make it harder to train models that remain reliable in practical deployment.

At the annotation level, Choi et al. pointed out that producing pixel-level labels for large aerial power line datasets is difficult and that the performance of supervised methods is closely tied to labeling cost [11]. Therefore, semi-supervised learning (SSL) becomes a promising solution because it allows a detector to learn from a few labeled subsets together with a much larger pool of unlabeled field images [12]. Some studies have researched SSL to show the effectiveness of the methods [13,14]. However, the conventional SSL often relies on biased risk estimates and unverified distributional assumptions, such as the cluster or smoothness assumptions, which can lead to models performing worse than simple supervised baselines. Schmutz et al. proposed debiased SSL (DeSSL), a framework that adds a debiasing term to nullify the inherent bias in the risk estimator under the standard missing completely at random (MCAR) assumption [15]. This approach provides essential theoretical guarantees for consistency, calibration, and safety, ensuring the algorithm remains reliable even when traditional geometric data assumptions are violated.

In this work, we aim to achieve efficient component detection, which is a foundational step towards the ultimate goal of fault detection. We propose a framework to conduct drone-based power line inspection. The framework consists of an SSL approach and hardware–software (HW-SW) co-optimization techniques for efficient deployment on resource-constrained edge devices. In this paper, the improved version of the edge-AI deployment score is proposed, which is named the generalized edge-AI deployment score (GEADS). The SSL can help to solve the labeling cost problem, while the edge-optimization technique and the GEADS can help the engineer to develop the most balanced performance for drone-based power line components that involves measuring key on-device metrics such as detection accuracy, inference time, memory footprint, power consumption, resource utilization, and model size. The main contributions of this paper are described as follows:

(1) A framework of effective and efficient drone-based power line inspection that involves an SSL approach and edge-device optimization.
(2) Comprehensive model and hardware performance evaluation to evaluate the SSL and edge-device optimization.
(3) The evaluation of GEADS to measure the balance performance between detection accuracy, inference time, and other hardware parameters.

This article is a revised and expanded version of our previous conference publication [16]. The enhancements of this paper include the inclusion of an SSL approach, the introduction of GEADS metrics, and the use of YOLOv9 and YOLOv9-Tiny in the context of hardware performance evaluation to increase the evaluation scenarios.

The remainder of this paper is organized as follows. Section 2 presents the research methodology, dataset preparation, an overview of the YOLO models, the SSL framework, the training and quantization procedures, deployment on edge devices, and the hardware performance testing setup. Section 3 presents the experimental results about the SSL method and edge-device evaluation. Section 4 discusses the findings of the research. Finally, Section 5 concludes the paper and outlines directions for future research.

## 2. Materials and Methods

**Figure 1:**
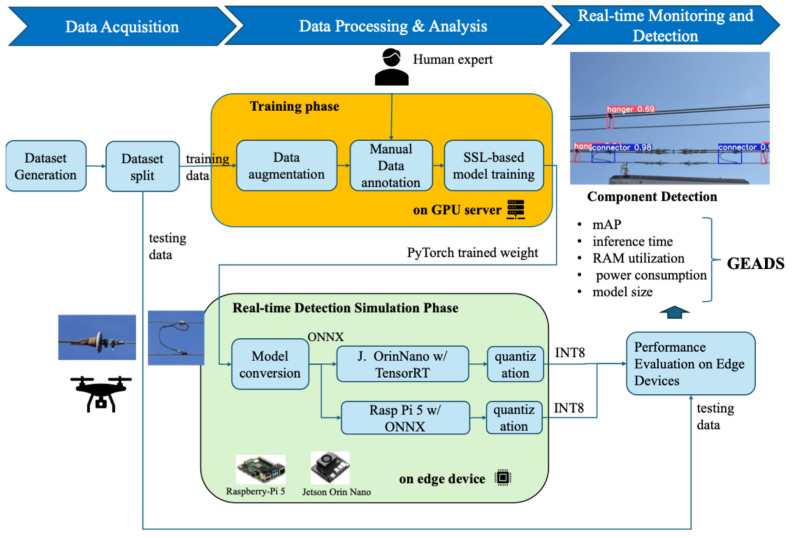
Proposed framework for edge-optimized SSL for power line component inspection.

The overall workflow of this study is illustrated in Figure 1. The process consists of several main stages: data collection and annotation, model training and selection, model quantization, accuracy performance evaluation, edge deployment, and hardware performance evaluation. Each stage is detailed in the following subsections.

### 2.1. Dataset Preparation

**Figure 2:**
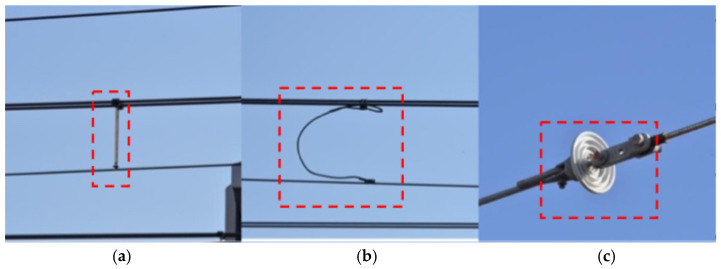
Dataset 1 consists of (**a**) hanger, (**b**) connector, (**c**) insulator.

Dataset 1 is our original dataset comprising 2400 images of power line equipment, with an additional 50 images reserved for test data. The images—featuring connectors, hangers, and insulators—are shown in Figure 2. Each image is manually annotated using the Visual Object Tagging Tool (VoTT) (v 2.2.0) [17]. Visual verification is also conducted to ensure bounding box accuracy. Prior to training, all images were uniformly resized to 640 × 640 pixels.

**Figure 3:**
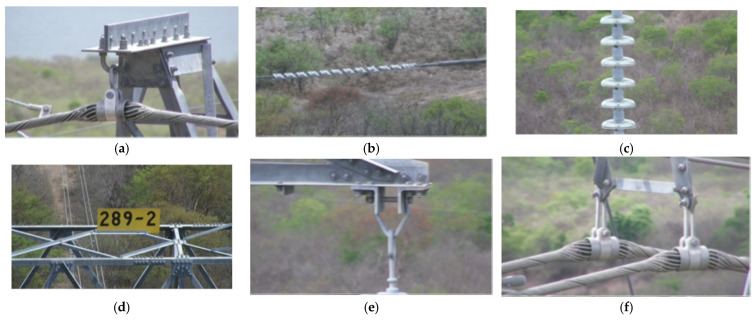
Representative images of dataset 2 consisting of (**a**) suspension clamp, (**b**) conductor, (**c**) insulator, (**d**) yoke plate, (**e**) armor rod, (**f**) spacer (from a total of 17 classes).

In this study, in addition to validation using dataset 1, we used the publicly available InsPLAD dataset [10], which labels power transmission line equipment for object detection, as dataset 2 to confirm the effectiveness of SSL under conditions with different data sizes and numbers of classes. The InsPLAD dataset contains UAV-based images for power line assets. The dataset includes variations in lighting conditions, object scale, viewpoint, camera distance, occlusion, cluttered background, and perspective distortion. It makes InsPLAD suitable for evaluating the performance of proposed method under practical inspections scenarios. The InsPLAD dataset consists of 10,561 images that are divided into 17 classes. Figure 3 shows 6 representative images of the InsPLAD dataset. In this paper, we evaluate the performance of supervised learning, semi-supervised learning, and DeSSL using dataset 1 and dataset 2. It is important to clarify that, although dataset 2 covers component defects, this study focuses only on component detection.

### 2.2. YOLO Model

The You Only Look Once (YOLO) family divides an input image into an S × S grid, where each cell simultaneously predicts bounding box coordinates, objectness scores, and class probabilities. This allows them to define object recognition as a single-stage, end-to-end regression task. Real-time inference and unified training are made possible by this design. YOLOv7 incorporates spatial pyramid pooling—cross-stage partial convolution (SPPCSPC) modules to increase the receptive field and speed inference—while introducing the extended efficient layer aggregation network (E-ELAN) backbone to maintain gradient flow [18]. YOLOv7 achieves higher frames per second (FPS) and better accuracy than previous YOLO versions while maintaining real-time performance, making it suitable for applications such as surveillance, autonomous driving, and industrial automation.

The tiny variant, i.e., YOLOv7-Tiny, follows the same lightweight philosophy—reducing network depth and width to remove redundant computations—making it well-suited for edge-device environments [19]. The modifications to create YOLOv7-Tiny involve reducing the model size and using a simplified architecture. It significantly reduces the parameter count to 6.2 million, compared to 36.9 million in YOLOv7. Compared to the other versions, the edge-optimized YOLOv7-Tiny uses the leaky rectified linear unit (ReLU) as the activation function, while other models use the sigmoid linear unit (SiLU) as the activation function. Throughout its development, YOLOv7-Tiny models have consistently aimed to balance speed, accuracy, and computational efficiency for surveillance, robotics, and IoT applications.

YOLOv9 is a one-stage object detector released in 2024 that primarily addresses the significant problem of information loss during feature transformation [20]. To combat this problem, the model introduces programmable gradient information (PGI), which utilizes an auxiliary reversible branch to retain critical data during training. The system’s backbone is built on the generalized efficient layer aggregation network (GELAN), combining efficient layer aggregation with flexible computational blocks to optimize speed and accuracy. These architectural innovations allow YOLOv9 to achieve superior results when trained from scratch, outperforming many models that rely on large-scale pre-training. Ultimately, YOLOv9 sets a new standard for real-time detection by ensuring that deep layers receive reliable gradients for effective parameter updates.

YOLOv9-Tiny (also referred to as YOLOv9-T) is the most compact variant, designed specifically to provide high-precision object detection for extreme resource-constrained environments [20]. With a small footprint of just 2.0 million parameters, this lightweight model achieves an impressive 38.3% average precision on the MS COCO benchmark. It utilizes the versatile GELAN architecture, which allows the model to maintain high parameter utilization without the need for complex depth-wise convolutions. The integration of PGI is particularly beneficial for this variant, as it allows the shallow network to benefit from auxiliary supervision without losing essential semantic information. Processing at only 7.7 GFLOPs, YOLOv9-Tiny represents a significant performance leap for edge devices compared to previous generations of “tiny” detectors. The summary of YOLOv7, YOLOv7-Tiny, YOLOv9, and YOLOv9-Tiny is shown in Table 1.

### 2.3. Model Training and Selection

Four YOLO variants (YOLOv7, YOLOv7-Tiny, YOLOv9, and YOLOv9-Tiny) are evaluated. All models were trained for 100 epochs with a batch size of 16. While these foundational settings were consistent, the core hyperparameters for the YOLOv7 and YOLOv9 series differed significantly, as detailed in Table 2. The YOLO v7 model is trained using the Adam optimizer, with an initial learning rate of 0.001 and final learning rate factor (lrf) of 0.1, resulting in a final learning rate of 1.0 × 10^−3^. The YOLOv9 model was trained using the SGD optimizer with an initial learning rate (lr0) of 0.01 and a final lrf of 0.01, resulting in a final learning rate of 1.0 × 10^−4^. These differences, particularly in the choice of optimizer and loss function gains, reflect their distinct architectural designs and optimization strategies.

### 2.4. Semi-Supervised Learning

The objective of standard SSL is to leverage information from the unlabeled data distribution p(x) to enhance a model’s predictive performance beyond what is achievable with limited labeled data. Typically, these methods aim to minimize a modified empirical risk estimator of the following form (1):

(1) $${\hat{R}}_{SSL}\left(\theta \right)=\frac{1}{n_{l}}\;\sum_{i\in L}L\left(\theta ;x_{i},y_{i}\right)+\frac{\lambda }{n_{u}}\;\sum_{j\in U}H\left(\theta ;x_{j}\right)$$

where *L* is the supervised loss on labeled samples L and *H* acts as an unsupervised surrogate term—such as entropy or consistency loss—weighted by a scalar *λ* on unlabeled samples U. $x_{i}$ and $y_{i}$ denote the *i*-th labeled sample and its corresponding ground-truth, respectively. $x_{j}$ denotes the *j*-th unlabeled sample. However, a significant drawback of this approach is that the resulting risk estimate is biased, even asymptotically, meaning $E[{\hat{R}}_{SSL}\left(\theta \right)]\neq R(\theta )$. This bias necessitates reliance on unverified distributional assumptions, such as the cluster or smoothness assumptions, which can cause performance to degrade if they do not hold.

To overcome these challenges, DeSSL was proposed by Schmudtz et al. [15], which incorporates a debiasing term to annul the inherent bias as presented by (2).

(2) $${\hat{R}}_{DeSSL}\left(\theta \right)=\frac{1}{n_{l}}\;\sum_{i\in L}L\left(\theta ;x_{i},y_{i}\right)+\frac{\lambda }{n_{u}}\;\sum_{j\in U}H\left(\theta ;x_{j}\right)-\frac{\lambda }{n_{l}}\;\sum_{i\in L}H\left(\theta ;x_{i}\right)$$

By subtracting the surrogate term evaluated on labeled data, DeSSL provides an unbiased estimator of the true risk under the missing completely at random (MCAR) assumption. This framework allows the model to benefit from the variance reduction properties of unlabeled data while maintaining essential theoretical guarantees for consistency, calibration, and safety, ensuring it never performs worse than the supervised baseline. In this work, we evaluate the performance of standard SSL and DeSSL with datasets 1 and 2 that represent two datasets with different numbers of samples and classes.

Although DeSSL was originally proposed for image classification, in this study it was adapted to YOLO-based object detection by applying the debiasing concept to the full YOLO detection loss rather than only to the classification output. Specifically, the YOLO detection loss consists of bounding-box regression loss, objectness loss, and classification loss. Therefore, when pseudo-labels are used as training targets, the unsupervised and debiasing terms affect both localization and class prediction.

For each mini-batch, images were randomly divided into labeled and unlabeled subsets using an image-level mask ratio. For images selected as unlabeled, the ground-truth annotations were removed from the supervised loss calculation. The model predictions were then processed by non-maximum suppression with an IoU threshold of 0.6, and detections with confidence scores higher than 0.95 were retained as pseudo-labels. Each pseudo-label was converted into YOLO training format, consisting of the image index, predicted class ID, and normalized bounding-box coordinates.

The resulting pseudo-labels were separated according to whether they originated from unlabeled or labeled images. Pseudo-labels from unlabeled images were used to compute the unsupervised detection loss. In the DeSSL setting, pseudo-labels generated from labeled images were additionally used to compute a debiasing loss, which was subtracted from the unlabeled pseudo-label loss. Thus, the final DeSSL-YOLO objective was implemented as a supervised YOLO detection loss plus a weighted debiased pseudo-label loss.

### 2.5. Deployment on Edge Devices

#### 2.5.1. Deployment on Jetson Orin Nano

Jetson Orin Nano is an edge device produced by Nvidia. It features a 1024-core NVIDIA Ampere GPU with 32 Tensor cores, an ARM Cortex-A78AE 6-core CPU, and 8 GB of LPDDR5 memory [21]. In this study, the YOLOv7, YOLOv7-Tiny, YOLOv9, and YOLOv9-Tiny models were implemented and optimized for deployment on the Jetson Orin Nano using the TensorRT (v 8.5.2.2) framework. TensorRT is an optimization tool for deep learning models on NVIDIA GPUs [22]. To meet real-time performance requirements on edge devices, model quantization was applied, converting the original full-precision (FP32) YOLO model to a lower-precision format (INT8). The trained weight files for YOLO were first converted to ONNX format. Then, the ONNX format files were converted to TensorRT using the TensorRT-For-YOLO-Series repository. This optimization significantly reduced model size and computational load, improving inference speed and reducing memory usage while maintaining acceptable accuracy.

Following the optimization, the hardware performance of the quantized YOLO model was evaluated on the Jetson Orin Nano. The evaluation metrics included inference time, memory usage, and power consumption, critical indicators for assessing the feasibility of edge AI. The Python time module was used to measure processing time. Processing time measured only the time taken for detection; code such as image loading was excluded from the measurement. The tegrastats command, a system monitoring tool provided by NVIDIA for Jetson platforms, was used to measure power consumption and memory usage on the Jetson device. The tegrastats command was used to obtain data such as power consumption and memory usage every 500 milliseconds (ms), and the average of this data was used as the final result. The detailed specifications and configurations are shown in Table 3.

#### 2.5.2. Deployment on Raspberry Pi 5

The Raspberry Pi (Raspberry Pi Foundation) is a low-cost, credit-card-sized single-board computer that is widely used for education, prototyping, and edge AI. A Broadcom BCM2712 SoC with a quad-core ARM Cortex-A76 CPU (up to 2.4 GHz), up to 8 GB LPDDR4X RAM, and a Video Core VII GPU (20) power the Raspberry Pi 5. It is one of the popular platform alternatives for deploying optimized deep-learning models at the edge due to its compact form factor, affordability, and moderate computing capabilities. A specific software configuration was established on a Raspberry PI for this research, as presented in Table 3.

Model quantization is used to reduce the inference time and resource consumption on platforms with limited resources, such as the Raspberry Pi. The Open Neural Network Exchange (ONNX) ecosystem was selected for this endeavor because of its notable benefits in terms of tooling, performance, and interoperability [23]. ONNX serves as a universal bridge between different deep learning frameworks, enabling us to train models in rich environments like PyTorch and deploy them seamlessly on devices that only require the lightweight ONNX Runtime engine. ONNX Runtime (version 1.8.1) is utilized to apply dynamic INT8 quantization to FP32-trained YOLO models [18].

The initial step in the quantization process was to use the export.py script with the --dynamic flag enabled to export the learned FP32-precision PyTorch models (.pt) to the common ONNX format. The quantize_dynamic function from the ONNX Runtime library is then used to apply post-training dynamic quantization to these ONNX models. It dynamically quantizes the activations during inference while converting the model’s weights to the INT8 data type to minimize file size. This approach yields an optimized INT8 ONNX model ready for efficient execution on the edge device, without requiring a calibration dataset.

Three primary steps comprised our strategy for implementing the models on the Raspberry Pi. Initially, the quantized INT8 ONNX models were transferred from the development PC to the device. Second, a Python virtual environment is created, and the libraries indicated in Table 3 are installed to set up the software environment. Finally, a Python script was implemented to manage the inference pipeline, which included pre-processing the input image, executing the model, and post-processing the output with non-maximum suppression (NMS) to obtain the final detections.

Every YOLO model is assessed on the Raspberry Pi 5 to thoroughly compare their practical performance on the embedded platform. Four important hardware-related characteristics were measured: power consumption, average inference time, memory use, and model size. A standardized methodology was employed for each measurement to ensure accuracy and reproducibility. The average inference time was measured using Python’s time.perf_counter() function for high precision. The model with a single warm-up inference was first used to get a stable result. Subsequently, the inference loop was run 50 times, and the mean processing time was calculated. Memory utilization was monitored using the psutil library. An external USB power meter, connected in series between the power source and the Raspberry Pi 5, was used to measure power consumption. While the model was continuously performing inference, five separate readings were taken from the power meter and the average was calculated to ensure a reliable, representative measurement of the power draw in watts (W).

### 2.6. Evaluation Metrics

#### 2.6.1. Model Accuracy Evaluation

We evaluate the performance of the deep learning model with mean average precision (mAP) and F1-score. mAP is a metric that evaluates an object detection model’s overall performance. It is calculated as the average of the area under the precision–recall curve (AP: average precision) for each class. mAP is shown by (3).

(3) $$\mathrm{mAP}=\frac{1}{N}\sum_{i=1}^{N}{\mathrm{AP}}_{i}$$

Here, N is the total number of classes and ${\mathrm{AP}}_{i}$ represents the area under the precision–recall curve for the *i*-th class. mAP comprehensively considers the balance between precision and recall at different confidence thresholds, with higher values indicating better overall model performance [9].

The F1-score is the harmonic mean of precision and recall and is a performance evaluation metric that considers both precision and recall. F1-score is shown by (4).

(4) $$F1\text{-}\mathrm{score}=2\times \frac{\mathrm{Precision}\times \mathrm{Recall}}{\mathrm{Precision}+\mathrm{Recall}}$$

A high F1-score indicates balanced detection performance with few false positives and false negatives. The closer the F1-score is to 1, the more effectively the model reduces both false positives (FPs) and false negatives (FNs) in a balanced manner.

#### 2.6.2. Edge-Deployment Evaluation (Proposed)

For edge-deployment evaluation, the improved version of the edge-deployment score is presented, which is the generalized edge-AI deployment score (GEADS). This metric is the improved version of EADS in our previous study [24]. Let *N* denote a deployment candidate, such as a model-device configuration. The general equation of GEADS $E\left(N\right)$ is presented in (5).

(5) $$E\left(N\right)=\prod_{k=1}^{m}{\tilde{x}}_{k}{\left(N\right)}^{w_{k}}$$

where ${\tilde{x}}_{k},\;m,\;{\;w}_{k}$ denote a normalized value of the *k*-th evaluation metric for candidate *N*, the total number of evaluation metrics used in evaluation, and the weighting coefficient assigned to the *k*-th metric that represents the priority of edge-AI application, respectively. The weighting coefficients satisfy (6).

(6) $$\sum_{k-1}^{m}w_{k}=1,\;w_{k}\geq 0\;$$

Therefore, the contribution of each metric is explicitly controlled according to its importance in the target application. To ensure a consistent interpretation in which larger normalized values always indicate better performance, benefit-type and cost-type metrics are normalized differently. For a benefit-type metric (larger value is preferrable), such as detection accuracy, the normalized value is given by (7).

(7) $${\tilde{x}}_{k}\left(N\right)=\frac{x_{k}(N)}{\underset{N}{\max}x_{k}(N)}$$

Whereas for the cost-type metric (smaller value is preferrable), such as inference time, resource utilization, or power consumption, the normalized value is defined as (8),

(8) $${\tilde{x}}_{k}\left(N\right)=\frac{\underset{N}{\min}x_{k}(N)}{x_{k}(N)}$$

With this normalization, all metrics are transformed into dimensionless quantities in the range between 0 and 1, where a larger value consistently indicates superior deployment suitability. This formulation avoids the scale distortion often associated with direct aggregation of heterogeneous metrics and is particularly well-suited for multiplicative score construction.

In this paper, five parameters (*m* = 5) are considered during edge-AI deployment, which are mAP, inference time, model size, RAM utilization, and power consumption. The general equation can be derived into (9),

(9) $$E\left(N\right)={\tilde{M}\left(N\right)}^{w_{1}}{\tilde{T}\left(N\right)}^{w_{2}}{\tilde{S}\left(N\right)}^{w_{3}}{\tilde{R}\left(N\right)}^{w_{4}}{\tilde{P}\left(N\right)}^{w_{5}}$$

where $\tilde{M}\left(N\right),\;\tilde{T}\left(N\right),\;\tilde{S}\left(N\right),\;\tilde{R}\left(N\right),\;\tilde{P}\left(N\right)$ denote the normalized mAP, inference time, model size, RAM utilization, and average power consumption for candidate *N* respectively. $w_{1},\;w_{2},\;w_{3},\;w_{4},\;w_{5}$ represent the assigned weight for the normalized mAP, inference time, model size, RAM utilization, and average power consumption, respectively. The weight is assigned based on the requirement of the application. In this paper, the highest priority is assigned for mAP@0.5 ($w_{1}=4/9)$, the second highest to time inference ($w_{2}=2/9)$, and the least priority given to model size, RAM utilization, and average power consumption equally ($w_{3},{\;w}_{4},{\;w}_{5}=1/9)$. These weights satisfy $\sum w_{i}=1.$ The largest weight is assigned to mAP@0.5 because detection accuracy is the primary requirement in power line inspection. Inference time was assigned the second largest weight because real-time processing is important for edge-assisted inspection. A multiplicative aggregation was adopted because GEADS is intended to evaluate balanced edge-deployment suitability rather than performance in a single metric. Compared with a weighted-sum formulation, the multiplicative form behaves like a weighted geometric mean and penalizes configurations that perform poorly in any one important metric. This property is suitable for edge-AI deployment because a model with high accuracy but excessive latency, memory usage, model size, or power consumption may not be practical for deployment, while a very small and fast model with insufficient accuracy may also be unacceptable. In contrast, a weighted sum is fully compensatory, meaning that an extremely good value in one metric can offset a very poor value in another metric. The multiplicative formulation reduces this compensation effect and favors models that maintain a more balanced performance profile across accuracy and deployment-efficiency metrics. Each normalized metric is defined in (10)–(14).

(10) $$\tilde{M}\left(N\right)=\frac{mAP(N)}{\underset{N}{\max}\;mAP(N)}$$

(11) $$\tilde{T}\left(N\right)=\frac{\underset{N}{\min}\;T_{inf}(N)}{T_{inf}(N)}$$

(12) $$\tilde{S}\left(N\right)=\frac{\underset{N}{\min}\;S_{mod}(N)}{S_{mod}(N)}$$

(13) $$\tilde{R}\left(N\right)=\frac{\underset{N}{\min}\;R(N)}{R(N)}$$

(14) $$\tilde{P}\left(N\right)=\frac{\underset{N}{\min}\;P(N)}{P(N)}$$

where $mAP\left(N\right),\;{\;T}_{inf}\left(N\right),\;S_{mod}\left(N\right),\;R\left(N\right),\;P\left(N\right)$ denote the mAP, inference time, model size, RAM utilization, and average power consumption for candidate *N* respectively.

The proposed GEADS metric improves EADS in one aspect which is the use of metric-level normalization before aggregation. In the original EADS formulation, raw performance indicators are first combined using weighted multiplicative aggregation and then scaled using min–max scaling [24]. In the proposed GEADSS, it is conducted in a different order. Each raw metric is first normalized to a dimensionless score, so that a higher value indicates better performance. GEADS is then calculated by applying weighted multiplicative aggregation to these normalized scores. This difference in normalization order is important. Since EADS aggregates raw metrics before normalization, the intermediate EADS value depends on the physical units used at inference time, for memory usage, and for power consumption. For example, changing inference time from milliseconds to seconds changes the raw EADS value, although the final scaled EADS ranking may remain unchanged if all scenarios are converted consistently. GEADS avoids this issue by normalizing each metric before aggregation. As a result, all inputs to the multiplicative aggregation are dimensionless and have the same optimization direction. Therefore, GEADS may produce a similar ranking to EADS’s for a set of scenarios. However, GEADS provides a consistent and more interpretable metric score compared to the EADS, which will be necessary in considering the recommended deployment scenario.

## 3. Results

In this section, the evaluation results are presented. The results consist of the SSL evaluation (on datasets 1 and 2) and the edge-optimized implementation that is evaluated using the proposed GEADS metrics.

### 3.1. SSL Evaluation

#### 3.1.1. Evaluation on Dataset 1

**Figure 4:**
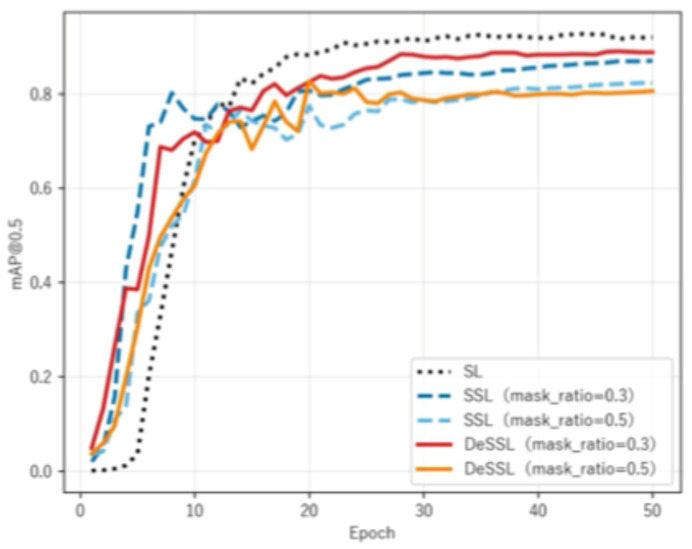
Accuracy evaluation of SSL on dataset 1.

Firstly, SSL is evaluated on our original dataset, dataset 1. The supervised learning (SL), standard SSL, and DeSSL methods are evaluated in this simulation. For the SSL method, mask ratios of 0.3 and 0.5 are employed in the simulation. A mask ratio of 0.3 means that only 70% of the training data are manually annotated, while the remaining 30% are not. The SSL evaluation parameters are shown in Table 4. In this simulation, we evaluate mAP when the intersection over union (IoU) threshold is 0.5 (mAP@0.5). In this case, the IoU ≥ 0.5 is considered a true positive, and the rest are considered false positives. All of the detection results are based on a single training run for each experimental configuration. Therefore, the reported mAP@0.5 and F1-score should be interpreted as single run-point estimates rather than performance over multiple random seeds. Figure 4 shows the mAP@0.5 vs. epoch performance. It shows that the mAP@0.5 performance of the three methods increases significantly from the first epoch until the 20th epoch. Finally, all of them achieve saturated performance at epoch 50.

**Figure 5:**
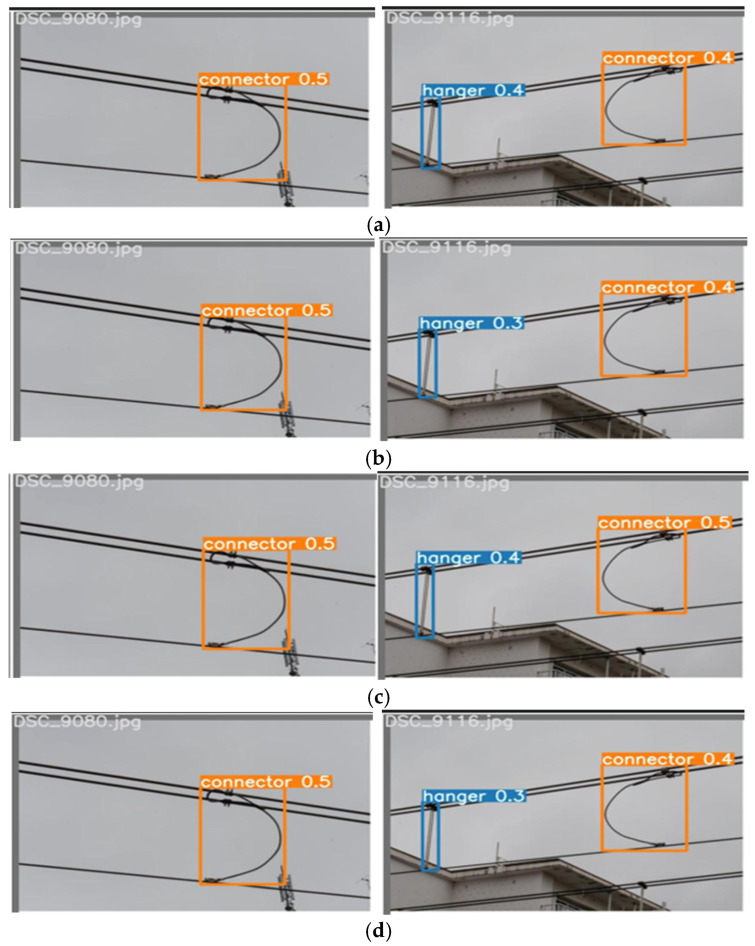
Power Component Line Prediction with dataset 1 using (**a**) SSL mask ratio 0.3, (**b**) SSL mask ratio 0.5, (**c**) DeSSL mask ratio 0.3, (**d**) DeSSL mask ratio 0.5.

Table 5 shows the summary of the F1-score and mAP@0.5 performance of every method at epoch 50. The DeSSL method achieves higher mAP@0.5 and F1-score than the standard SSL with mask ratios of 0.3 and 0.5, while, as expected, it achieves lower mAP@0.5 than the SL. For example, at a mask ratio of 0.3, DeSSL achieves mAP@0.5 of 0.906, which is only slightly lower than SL’s mAP@0.5 of 0.924. It means the DeSSL method can help to reduce the labeling burden while only suffering a slight decrease in detection performance. Table 6 shows the mAP@0.5 for SL, SSL, and DeSSL across all components in the dataset. The three methods exhibit a pattern similar to the general performance shown in Table 5. Figure 5 shows bounding-box visualizations of the detection performance of the SSL and DeSSL models. From Figure 5, it can be seen that DeSSL achieves slightly higher accuracy than the standard SSL. For example, the detection of the connector component in the right-side figure can be achieved with a 0.5 confidence score by DeSSL (Figure 5c), compared to 0.4 by SSL (Figure 5a).

#### 3.1.2. Evaluation on Dataset 2

**Figure 6:**
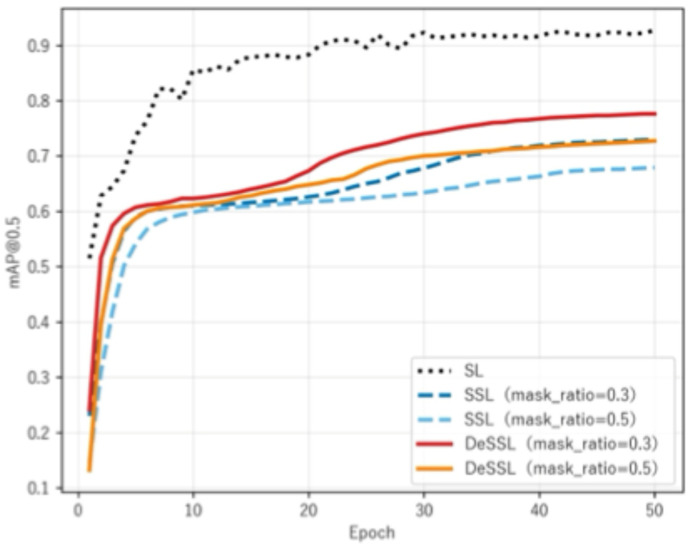
Accuracy evaluation of SSL on dataset 2.

Secondly, the SSL is evaluated on the InsPLAD dataset, which is dataset 2. The supervised learning (SL), standard SSL, and DeSSL methods are evaluated in this simulation. For the SSL method, mask ratios of 0.3 and 0.5 are employed in the simulation. The SSL evaluation parameters are shown by Table 7. Figure 6 shows the mAP@0.5 vs epoch performance. It shows that the mAP@0.5 performance of the three methods increases significantly from epoch 1 to epoch 10, and all achieve saturated performance at epoch 50.

Table 8 shows the summary of F1-score and mAP@0.5 performance of every method at epoch 50. The DeSSL method achieves higher mAP@0.5 and F1-score than the standard SSL at mask ratios of 0.3 and 0.5, while, as expected, it achieves lower mAP@0.5 than the SL. For example, at a mask ratio 0.3, DeSSL achieves mAP@0.5 of 0.768, which is only slightly higher than the standard SSL mAP@0.5 of 0.751. However, Table 8 also shows a larger decrease in mAP@0.5 between the DeSSL and SL methods. Table 9 shows the mAP@0.5 for SL, SSL, and DeSSL across all components in the dataset. For this paper, an evaluation of the 17 classes was conducted. The three methods exhibit a similar pattern to the general performance shown in Table 6. However, for some components with a smaller amount of data, such as spacer, vibration damper, and jumper [10], there is a significant decrease in mAP@0.5 performance of the DeSSL method compared to the SL method. It can be understood that the significant decrease in mAP@0.5 performance between DeSSL and SL methods occurs due to the detection performance of components with a smaller amount of data.

**Figure 7:**
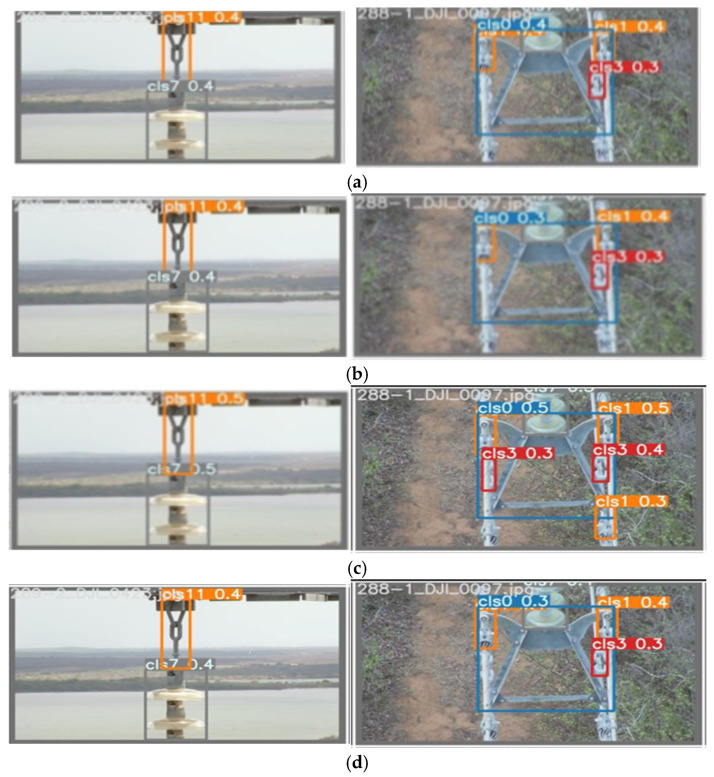
Power component line prediction with dataset 2 using (**a**) SSL mask ratio 0.3, (**b**) SSL mask ratio 0.5, (**c**) DeSSL mask ratio 0.3, (**d**) DeSSL mask ratio 0.5.

Figure 7 shows the bounding box visualizations that show the detection performance of SSL and DeSSL models. From Figure 7, it can be seen that the DSSL achieves slightly higher accuracy than the standard SSL. For example, the detection of some components in Figure 7c (DeSSL 0.3) achieves a higher confidence score compared the results shown by Figure 7a (SSL 0.3). On other hand, it can also be noticed that the SSL with a mask ratio of 0.3 (Figure 7a,c) achieves a higher confidence score compared to the SSL with a mask ratio of 0.5 (Figure 7b,d).

### 3.2. Evaluation on Edge Devices

In this section, the evaluation on edge devices is conducted in terms of mAP@0.5, memory usage (MB), model size (MB), power consumption (W), and average inference time (ms). At the end, the GEADS is calculated to understand the configuration that achieves the most balanced performance with all required metrics. There are 16 configurations that consist of different evaluation platforms (Raspberry Pi and Jetson Orin Nano), quantization modes (floating-point 32-bit (FP32) and integer 8-bit (INT8)), and YOLO model versions (YOLOv7, YOLOv7-Tiny, YOLOv9, and YOLOv9-Tiny).

Table 10 shows the mAP@0.5 performance of different YOLO versions with different quantization modes. The mAP@0.5 comparison shows that INT8 quantization results in a slight but consistent decrease in detection accuracy across all models. YOLOv9, the top-performing model, experienced a drop in mAP@0.5 from 0.914 (FP32) to 0.898 (INT8). YOLOv7 saw its mAP@0.5 decrease from 0.898 to 0.893. The lightweight models showed a similar trend, with YOLOv9-Tiny dropping from 0.901 to 0.882 and YOLOv7-Tiny from 0.888 to 0.876. This demonstrates that, while quantization reduces the model’s precision, its overall impact on mAP@0.5 is marginal, with the performance drop typically less than 2% across most models.

To systematically evaluate the effect of INT8 quantization on detection performance, the absolute and relative losses of mAP@0.5 and F1-score are presented in Table 11. The absolute mAP@0.5/F1-score loss was calculated as the difference of mAP@0.5/F1-score between FP32 and INT8 of the same YOLO model. The relative mAP@0.5/F1-score loss was calculated as the absolute loss divided by the FP32 baseline performance. This comparison allows us to determine whether INT8 quantization achieves deployment efficiency without excessive degradation in detection accuracy. For example, YOLOv7-Tiny INT8 only causes absolute mAP@0.5 loss of 0.012, relative mAP@0.5 loss of 1.351%, absolute F1-score loss of 0.008, and relative F1-score loss of 0.913%. It means the YOLO INT8 model retains about 98.649% of the FP32 maP@0.5 performance and about 99.087% of the FP32 F1-score performance. From the results shown in Table 11, it can be clarified that the INT8 quantization provides deployment advantages with limited accuracy degradation.

In terms of model size, INT8 quantization yields a significant reduction in model size, with file sizes shrinking by approximately 4 times on average. For instance, YOLOv7 shrinks from 139.32 MB to 35.24 MB. This reduction is critical for deployment on edge devices with limited storage. Memory usage also decreases substantially, with INT8 models consuming roughly half the RAM of their FP32 counterparts during inference. For instance, the INT8 version of the YOLOv7 model consumes 68.53 MB, which is significantly lower than the FP32 model’s 159.62 MB.

The largest improvement is observed in inference speed. INT8 quantization provides a significant acceleration across all models. The full-sized YOLOv7 model’s inference time improved by 3.9 times (from 1702.78 ms to 434.32 ms) on Raspberry Pi 5 implementation and 1.73 times (from 549.50 ms to 318.41 ms) on Jetson Orin Nano implementation. On the other hand, YOLOv9 achieved a 3.3× speedup (from 3917.08 ms to 1198.6 ms). Among the lightweight models, YOLOv7-Tiny emerged as the top performer for real-time applications, achieving the fastest inference time of just 115.94 ms (~8.6 FPS) in its INT8 version.

Power consumption remains relatively stable, with the INT8 models generally operating in a similar or slightly lower power band to their FP32 counterparts. Notably, the quantized lightweight models, especially YOLOv7-Tiny (8.744 W), show the lowest power draw, making them highly efficient for battery-powered or power-sensitive applications.

Finally, the GEADS is used to determine which configuration achieves the most balanced performance across detection accuracy, inference time, RAM utilization, power consumption, and model size. The GEADS is calculated using Equation (9). Quantized YOLOv7-Tiny (INT8) implementation on the Raspberry Pi shows the best score of 0.657 due to an acceptable accuracy result, outstanding inference time, and other hardware performance results. YOLO-v9 (FP-32) implementation on the Jetson Orin Nano shows the lowest scores, 0.157, due to the very large value of inference time, relatively large value of power consumption, and model size.

To further examine the best configuration, a sensitivity analysis was conducted under three alternative weighting scenarios. The first alternative scenario used equal weights for all metrics. The second alternative scenario emphasized accuracy by increasing the mAP@0.5 weight. The third alternative scenario emphasized deployment efficiency by increasing the weights for latency, RAM utilization, model size, and power consumption. The weight details are presented in Table 12. This analysis was performed using the same experimental results as shown in Table 10.

**Figure 8:**
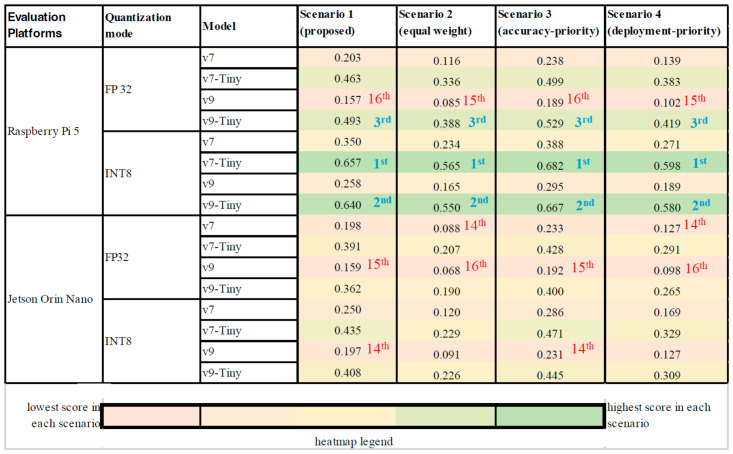
Sensitive Analysis of GEADS weight.

Figure 8 shows the sensitivity analysis of GEADS under four different weighting scenarios. The results show a near-consistent ranking across all scenarios. It shows that the top three rankings are consistent across all scenarios. The YOLOv7-Tiny with INT8 on the Raspberry Pi 5 achieves the highest score for all scenarios, followed by YOLOv9-Tiny INT8 on the Raspberry Pi 5 and YOLOv9-Tiny FP32 on the Raspberry Pi 5, respectively. However, we can see that their score decreases in scenarios 2 and 4, where the weight on accuracy is lower than in scenarios 1 and 3. It means the top 3 scenarios have strong accuracy performance. The interesting pattern can be noticed for the bottom 3, where YOLOv9 FP32 on the Jetson Orin Nano and YOLOv9 FP32 on the Raspberry Pi occupy positions 15 and 16 alternately in all four scenarios. It means that final GEADS can also be influenced by weighting configurations. It is proven by consistent ranking between scenarios 1 and 3, where more priority is given to accuracy. However, scenario 4 which emphasizes deployment shows slightly different ranking to scenarios 1 and 3.

**Figure 9:**
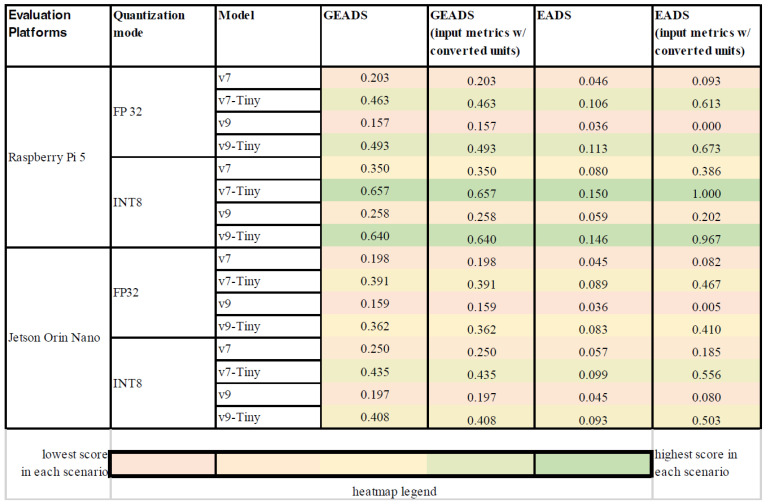
Sensitive Analysis of GEADS vs. EADS with different units of input metrics (inference time (µs), RAM utilization (KB), file size (KB), power consumption (mW)).

**Figure 10:**
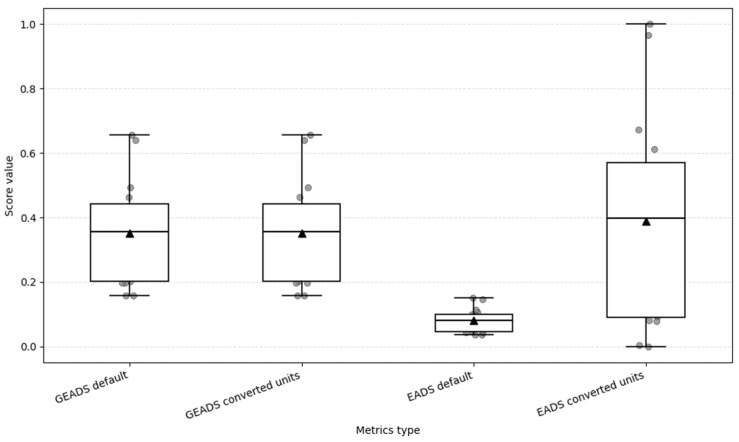
Boxplot Analysis of GEADS vs. EADS with different units of input metrics (inference time (µs), RAM utilization (KB), file size (KB), power consumption (mW)). (gray circle: individual deployment-scenario score, black triangle: mean value).

Figure 9 shows the comparison of the proposed GEADS and the conventional EADS in two different conditions. The first condition is GEADS and EADS using the default metrics unit as shown in Table 10. The second condition is GEADS and EADS using the converted units, i.e., inference time (µs), RAM utilization (KB), file size (KB), and power consumption (mW). The converted units are intended to evaluate the change in metric score across different metric unit conditions. The heatmap in Figure 9 shows that the GEADS metric score remains unchanged in both scenarios, while the EADS shows a significant difference in metrics scores in both scenarios. The statistical characteristics of GEADS and EADS are shown in the boxplots in Figure 10. It shows that the GEADS distribution remains identical before and after unit conversion, while the EADS distribution changes substantially. This indicates that GEADS is more stable at the numerical-score level because each metric is normalized before aggregation. In contrast, EADS aggregates raw unit-dependent values before normalization, making its score distribution sensitive to the selected measurement units. However, the ranking remains similar because the unit conversion is applied consistently across all deployment scenarios. From these results, it is clear that GEADS is superior to EADS not because it changes the final ranking but because its score distribution remains invariant under unit conversion, making it more interpretable and statistically stable as a deployment suitability metric. The numerical score is also important because it indicates the magnitude of the deployment advantage. A small score difference between the first- and second-ranked scenarios suggests that both configurations provide comparable deployment suitability, whereas a large difference indicates a clearer advantage.

Finally, these on-device evaluations demonstrate that INT8 quantization is a highly effective and essential optimization strategy. It significantly improves inference speed and reduces the resource consumption, transforming even large models into more viable options for edge deployment. The implementation on the Raspberry Pi 5 with quantized YOLOv7-Tiny INT8 offers the best overall package, delivering the fastest inference rate while consuming low power.

## 4. Discussion

In this paper, there are two different evaluations. In the first evaluation, the learning effectiveness of the SSL method under limited data was evaluated. In the second evaluation, the edge deployment was evaluated. In the first evaluation, YOLOv7 was selected for the SSL evaluation because this part of study aimed to evaluate the effectiveness of SSL approach under the limited labeled data, rather than to optimize the final edge-deployment model. The full YOLOv7 model has a larger network capacity, with more parameters and computational operations [18], enabling it to learn richer visual representations from both labeled and unlabeled images. This is important in SSL because the effectiveness of pseudo-label-based models depends strongly on the reliability of model predictions on unlabeled samples [25]. In contrast, YOLOv7-Tiny and other lightweight methods were mainly evaluated in the edge deployment stage because their lightweight architecture is more suitable for resource-constrained devices [19].

Regarding the dataset used, it is important to clarify that the generalizability of the proposed framework is affected by the characteristics of datasets 1 and 2. Dataset 1 contains 2400 images, which is relatively small for deep-learning-based object detection. This limitation may affect the trained model’s ability to generalize across a wide range of object appearances, backgrounds, and environmental conditions encountered in practical power line inspection. To partially address this issue, dataset 2 was used, as it contains UAV-based power line inspection images captured in real-world, uncontrolled environments. Dataset 2 includes variation in object scale, camera viewpoint, perspective distortion, occlusion, cluttered background, and lighting conditions. Therefore, dataset 2 provides a more realistic assessment of the proposed framework under practical visual variations. However, dataset 2 does not fully cover all possible weather conditions that may occur in a real-world setting. As a result, the performance demonstrated in this study should be interpreted as performance with the visual variations represented in the evaluated datasets.

In the first SSL evaluation, it can be noticed that SSL and DeSSL with a mask ratio of 0.3 achieve higher mAP@0.5 and F1-score than SSL and DeSSL with a mask ratio of 0.5. They perform as expected since the number of unlabeled data with a mask ratio of 0.5 is higher than that of a mask ratio of 0.3. It can also be noticed in both dataset 1 and dataset 2 that DeSSL achieves slightly higher accuracy than the standard SSL method. However, in dataset 2, when the mask ratio was increased, the accuracy degradation of DeSSL was smaller than that of SSL, and the stability under the condition of a high unlabeled ratio was confirmed. Specifically, when the SSL mask ratio was increased from 0.3 to 0.5 in dataset 2, mAP@0.5 dropped from 0.751 to 0.695, while DeSSL saw a smaller decrease from 0.768 to 0.750. In addition, when comparing DeSSL across datasets, mAP@0.5 decreases from 0.906 to 0.845 as the mask ratio increases from 0.3 to 0.5 in dataset 1, whereas the decrease is relatively small in dataset 2. From the above, the fact that the performance degradation of DeSSL was suppressed under the condition of a high unlabeled ratio is because DeSSL has the property of suppressing learning instability, and its effect may have more easily appeared in dataset 2, which is more complex due to a higher number of classes. It confirms the safety aspects of DeSSL that can still perform better at a high mask ratio. It means DeSSL can help reduce the burden of the labeling process without significantly sacrificing detection performance.

In the second evaluation, the evaluation on the edge device was conducted. Detection shows that the latest YOLOv9 model achieves the highest detection accuracy (mAP@0.5 0.914). The on-device tests on the Raspberry Pi 5 underscored a critical trade-off: larger, more accurate models like YOLOv9 are too slow for practical real-time applications, with inference times of nearly 4 s. In contrast, lightweight models are essential for deployment, with the quantized YOLOv7-Tiny emerging as the optimal choice due to its fast inference speed of 115.94 ms and efficient resource usage.

Furthermore, our evaluations confirmed that INT8 quantization is a highly effective optimization strategy, dramatically improving inference speeds by up to 4 times and significantly reducing memory usage, all while incurring only a minimal loss in detection accuracy. It can also be noted that the Jetson Orin Nano can help reduce inference time, but this comes at the cost of a larger model size and higher memory usage. In general, INT8 quantization also achieves lower power consumption than FP32. However, in some cases on the Jetson Orin Nano, an irregularity is observed where the power consumption for FP32 quantization is lower than that for INT8 quantization. It should be noted that the power consumption reported in this paper represents the average measured power during the inference execution window, not the total energy per inference. On the Jetson Orin Nano, INT8 TensorRT inference can execute faster but may also increase GPU utilization and clock activity during the measurement period, resulting in higher instantaneous average power than FP32 in some cases.

Ultimately, this work successfully demonstrates that cost-effective, real-time object detection for power line monitoring is achievable but requires careful model selection and optimization to balance accuracy with the computational limits of edge hardware. Therefore, our proposed GEADS can be one of the methods for selecting a configuration that achieves the most balanced performance. The GEADS evaluation shows that the implementation of quantized YOLOv7-Tiny (INT8) on a Raspberry Pi 5 achieved the highest GEADS = 0.657 (rank 1). This result reflects its overall trade-off among required metrics, i.e., detection accuracy, inference latency, RAM utilization, model size, and power consumption. However, the best GEADS achievement does not necessarily correspond to the best mAP@0.5 performance, which is the highest-priority metric in this paper. YOLOv7-Tiny INT8 achieves mAP@0.5 of 0.876, lower than YOLOv9 FP32 (mAP@0.5 of 0.914) and YOLOv9 INT8 (mAP@0.5 of 0.898). We understand that there is no formal mAP@0.5 performance standard for power line inspection in practice. However, from the comprehensive review conducted by Faisal et al. [26], we understand that the current high-performance systems typically aim for mAP@0.5 scores above 85% for larger components, while scores for very small objects remain lower. Based on previous studies, it means that the mAP@0.5 achieved by YOLOv7-Tiny (mAP@0.5 of 0.876) is still acceptable for power line inspection in practice. Nevertheless, for the applications that prioritize maximum detection accuracy, the adjustment of mAP@0.5 weight to a higher value is required.

## 5. Conclusions

This study introduced SSL using YOLOv7 as the base detector and applied DeSSL to suppress learning bias, aiming to achieve both reduced labeling costs and maintained performance. Experimental results showed that, while the final accuracy difference between SSL and DeSSL was not significant, DeSSL tended to exhibit less performance degradation when the mask ratio was increased from 0.3 to 0.5. This suggests that DeSSL can suppress learning instability and mitigate performance degradation under conditions with a high proportion of unlabeled data. Furthermore, this study used a dataset where all data was labeled and simulated unlabeled data by treating some labels as unused during training. Mask ratios of 0.3 and 0.5 were benchmarks for reducing labeling workload by approximately 30% and 50%, respectively. Based on these findings, it can be concluded that DeSSL is a promising method for safely operating semi-supervised learning, as it is less likely to lead to performance degradation even as the amount of unlabeled data increases. In this research, a comprehensive performance evaluation of four modern YOLO models (YOLOv7, YOLOv7-Tiny, YOLOv9, and YOLOv9-Tiny) was conducted with a total of 16 configurations for the automated detection of power line equipment, focusing on their potential for deployment on resource-constrained embedded systems. The GEADS is also proposed to select the configuration that achieves the most balanced performance across the required parameters. The evaluation results show that the GEADS metric yields a more interpretable score than the traditional EADS method.

Future work will focus on deeper, hardware-specific optimization using an FPGA hardware accelerator. We will also explore deploying the top-performing models on different classes of embedded hardware to compare their performance. In this study, the dataset used also has limitations. For example, the utilized dataset does not cover different weather, such as sunny, cloudy, rainy, or foggy conditions. Therefore, the present evaluation mainly reflects robustness to uncontrolled visual variations which are supported by dataset 1 and dataset 2, including lighting, viewpoint, occlusion, distance, and background complexity. Therefore, a training regimen using a larger, more detailed weather-specific dataset, powered by a continuous data collection and augmentation pipeline, will be established to improve the detection model’s accuracy. Finally, all accuracy results in this study were obtained in single-run experiment settings. Multi-seed experiments might be required to confirm statistical consistency in future work.

## Figures and Tables

**Table 1:** YOLOv7, YOLOv7-Tiny, YOLOv9, and YOLOv9-Tiny Comparison.

Feature	YOLOv7	YOLOv7-Tiny	YOLOv9	YOLOv9-Tiny
Backbone	Extended-ELAN (E-ELAN), spatial pyramid pooling–cross-stage partial convolution (SPPCSPC)	Tiny-ELAN, Conv + Bn + Silu (CBS) layer, max pooling (MP) layer	Generalized efficient layer aggregation network (GELAN)	GELAN (small configuration)
Number of parameters	36.9 million	6.2 million	25.3 million (for YOLOv9-C)	7.1 million
Detection head	Anchor-based	IDetect detection head	Anchor-free features programmable gradient information (PGI) with an auxiliary reversible branch	Anchor-free
Use cases	Cloud/server-based or high-end edge-AI hardware	Resource-constrained edge devices	Industrial robots, autonomous driving	Resource-constrained edge devices requiring higher accuracy

**Table 2:** Hyperparameter Comparison.

Hyperparameter	YOLOv7 and YOLOv7-Tiny	YOLOv9 and YOLOv9-Tiny
Image Size	640 × 640	640 × 640
Batch Size	16	16
Epochs	100	100
Optimizer	Adam	SGD
Initial Learning Rate (lr0)	0.01	0.01
Final LR Factor (lrf)	0.1	0.01
Box Loss Gain (box)	0.05	7.5

**Table 3:** Evaluation Platforms’ Hardware and Software.

Specification	Evaluation Platform	
	Raspberry Pi 5	Jetson Orin Nano
CPU	1.5 GHz quad-core Cortex A-72	6-core Arm Cortex-A78AE
Hardware accelerator	-	GPU 1024-core Ampere
RAM	8 GB LPDDR4	8 GB 128-bit LPDDR5
Harddisk	microSDHC card 64 GB	microSDHC 64 GB
OS	Debian GNU/Linux 12	Jetpack 5.1.1 (Ubuntu 20.04)
Softwares	Python 3.11.2 ONNX Runtime 1.22.0 OpenCV-Python 4.11.0.86	Python 3.8.10 PyTorch 2.0.0 Torchvision 1.14 TensorRT 8.5.2.2

**Table 4:** SSL Evaluation on dataset 1.

Specification	Configured Value
Dataset	Original dataset
Number of classes	3
Amount of data	2400 images
Data proportion (training:validation:testing)	8:1:1
Method	SL, SSL, DeSSL
Model	YOLOv7
Mask ratio	0.3, 0.5
Epoch	50
Evaluation metrics	mAP@0.5, F1-score

**Table 5:** F1-score and mAP performance on dataset 1.

Method	Mask Ratio	F1-Score	mAP@0.5
SL		0.878	0.924
SSL	0.3	0.855	0.870
	0.5	0.838	0.825
DeSSL	0.3	0.872	0.906
	0.5	0.836	0.845

**Table 6:** mAP performance of component detection on dataset 1.

Class ID	Component	SL	SSL		DeSSL	
			Mask: 0.3	Mask: 0.5	Mask: 0.3	Mask: 0.5
cls0	hanger	0.912	0.831	0.712	0.863	0.755
cls1	connector	0.994	0.994	0.988	0.989	0.933
cls2	insulator	0.866	0.783	0.775	0.867	0.786

**Table 7:** DeSSL Evaluation on dataset 2.

Specification	Configured Value
Dataset	InsPLAD dataset [10]
Number of classes	17
Amount of data	10,561 images
Data proportion (training:validation:testing)	8:1:1
Method	SL, SSL, DeSSL
Model	YOLOv7
Mask ratio	0.3, 0.5
Epoch	50
Evaluation metrics	mAP@0.5, F1-score

**Table 8:** F1-score and mAP performance on dataset 2.

Method	Mask Ratio	F1-Score	mAP@0.5
SL	N/A	0.909	0.943
SSL	0.3	0.768	0.751
	0.5	0.739	0.695
DeSSL	0.3	0.769	0.768
	0.5	0.786	0.750

**Table 9:** mAP@0.5 performance on dataset 2.

Class ID	Component	SL	SSL		DeSSL	
			Mask: 0.3	Mask: 0.5	Mask: 0.3	Mask: 0.5
cls0	damper	0.972	0.957	0.956	0.964	0.950
cls1	insulator	0.992	0.968	0.961	0.984	0.978
cls2	spacer	0.961	0.759	0.439	0.556	0.705
cls3	tower	0.989	0.972	0.966	0.979	0.968
cls4	tower plate	0.972	0.605	0.523	0.721	0.752
cls5	stockbridge damper	0.996	0.995	0.995	0.995	0.995
cls6	grading ring	0.995	0.989	0.989	0.991	0.985
cls7	spacer damper	0.988	0.952	0.937	0.970	0.941
cls8	yoke plate	0.995	0.995	0.995	0.995	0.995
cls9	suspension clamp	0.996	0.975	0.985	0.980	0.971
cls10	tension clamp	0.956	0.848	0.827	0.855	0.824
cls11	armor rod	0.994	0.973	0.968	0.981	0.941
cls12	vibration damper	0.995	0.008	0.003	0.125	0.064
cls13	jumper	0.695	0.146	0.032	0.160	0.089
cls14	jumper clamp	0.682	0.421	0.182	0.473	0.413
cls15	ground wire	0.861	0.211	0.063	0.337	0.195
cls16	conductor	0.991	0.990	0.989	0.989	0.988

**Table 10:** The evaluation results on edge devices.

Evaluation Platforms	Quantization Mode	Model	mAP@0.5	Model Size (MB)	Memory Usage (MB)	Avg. Inference Time (ms)	Power Consumption (W)	GEADS	GEADS Rank
Raspberry Pi 5	FP 32	v7	0.898	139.32	159.62	1702.78	9.937	0.203	12
		v7-Tiny	0.888	23.03	34.84	206.76	10.807	0.463	4
		v9	0.914	193.68	223.14	3917.08	10.670	0.157	16
		v9-Tiny	0.901	10.27	32.38	252.14	10.564	0.493	3
	INT8	v7	0.893	35.24	68.53	434.32	10.425	0.350	9
		v7-Tiny	0.876	5.97	21.67	115.94	8.744	0.657	1
		v9	0.898	49.45	98.62	1198.60	10.790	0.258	10
		v9-Tiny	0.882	3.61	35.47	129.61	9.129	0.640	2
Jetson Orin Nano	FP32	v7	0.898	288	2707	549.50	3.440	0.198	13
		v7-Tiny	0.888	50	2380	86.20	1.950	0.391	7
		v9	0.914	433	3237	1121.42	3.480	0.159	15
		v9-Tiny	0.901	30	2625	115.61	3.470	0.362	8
	INT8	v7	0.893	129	2011	318.41	3.770	0.250	11
		v7-Tiny	0.876	22	2983	51.79	3.520	0.435	5
		v9	0.898	206	2449	668.03	3.790	0.197	14
		v9-Tiny	0.882	18	2201	87.95	3.690	0.408	6

**Table 11:** Accuracy loss evaluation due to quantization.

Model	FP32 mAP@0.5	INT8 mAP@0.5	Absolute mAP@0.5 Loss	Relative mAP0.5 Loss (%)	FP32 F1-Score	INT8 F1-Score	Absolute F1-Score Loss	Relative F1-Score Loss (%)
v7	0.898	0.893	0.005	0.557	0.884	0.878	0.006	0.679
v7-Tiny	0.888	0.876	0.012	1.351	0.876	0.868	0.008	0.913
v9	0.914	0.898	0.016	1.751	0.878	0.864	0.014	1.595
v9-Tiny	0.901	0.882	0.019	2.109	0.874	0.858	0.016	1.831

**Table 12:** Sensitivity Analysis Scenario.

Scenario	$w_{1}$	$w_{2}$	$w_{3}$	$w_{4}$	$w_{5}$
Scenario 1 (proposed)	0.444	0.222	0.111	0.111	0.111
Scenario 2 (equal weights)	0.200	0.200	0.200	0.200	0.200
Scenario 3 (accuracy-priority)	0.500	0.200	0.100	0.100	0.100
Scenario 4 (deployment-priority)	0.350	0.250	0.150	0.150	0.150

## Data Availability

The raw data supporting the conclusions of this article will be made available by the authors on request.
